# IFI27 is a potential therapeutic target for HIV infection

**DOI:** 10.1080/07853890.2021.1995624

**Published:** 2022-01-22

**Authors:** Huijuan Huang, Jiannan Lv, Yonglun Huang, Zhiyi Mo, Haisheng Xu, Yiyang Huang, Linghui Yang, Zhengqiu Wu, Hongmian Li, Yaqin Qin

**Affiliations:** a Department of Infectious Diseases, Guiping People’s Hospital, Guigping, Guangxi, China; b Department of Infectious Diseases, The Affiliated Nanning Infectious Disease Hospital of Guangxi Medical University and The Fourth People’s Hospital of Nanning, Nanning, Guangxi, China; c Department of Ophthalmology and Otorhinolaryngology, Guiping People’s Hospital, Guigping, Guangxi, China; d Department of Physical Examination Center, Guiping People’s Hospital, Guigping, Guangxi, China; e Department of Burn and Plastic Surgery, The People’s Hospital of Binyang County, Binyang, Guangxi, China; f Research Center of Medical Sciences, The People’s Hospital of Guangxi Zhuang Autonomous Region & Guangxi Academy of Medical Sciences, Nanning, Guangxi, China

**Keywords:** HIV, coexpression, LASSO regression, immune cell infiltration, IFI27

## Abstract

**Background:**

Therapeutic studies against human immunodeficiency virus type 1 (HIV-1) infection have become one of the important works in global public health.

**Methods:**

Differential expression analysis was performed between HIV-positive (HIV+) and HIV-negative (HIV-) patients for GPL6947 and GPL10558 of GSE29429. Coexpression analysis of common genes with the same direction of differential expression identified modules. Module genes were subjected to enrichment analysis, Short Time-series Expression Miner (STEM) analysis, and PPI network analysis. The top 100 most connected genes in the PPI network were screened to construct the LASSO model, and AUC values were calculated to identify the key genes. Methylation modification of key genes were identified by the chAMP package. Differences in immune cell infiltration between HIV + and HIV- patients, as well as between antiretroviral therapy (ART) and HIV + patients, were calculated using ssGSEA.

**Results:**

We obtained 3610 common genes, clustered into nine coexpression modules. Module genes were significantly enriched in interferon signalling, helper T-cell immunity, and HIF-1-signalling pathways. We screened out module genes with gradual changes in expression with increasing time from HIV enrolment using STEM software. We identified 12 significant genes through LASSO regression analysis, especially proteasome 20S subunit beta 8 (PSMB8) and interferon alpha inducible protein 27 (IFI27). The expression of PSMB8 and IFI27 were then detected by quantitative real-time PCR. Interestingly, IFI27 was also a persistently dysregulated gene identified by STEM. In addition, 10 of the key genes were identified to be modified by methylation. The significantly infiltrated immune cells in HIV + patients were restored after ART, and IFI27 was significantly associated with immune cells.

**Conclusion:**

The above results provided potential target genes for early diagnosis and treatment of HIV + patients. IFI27 may be associated with the progression of HIV infection and may be a powerful target for immunotherapy.

## Introduction

More than 30 years after the discovery that human immunodeficiency virus (HIV) is the causative agent of acquired immunodeficiency syndrome (AIDS), HIV remains a major challenge to global public health [1]. According to statistics, more than 36.9 million people were infected with HIV in 2018 [2]. Early receipt of antiretroviral therapy (ART) after a positive diagnosis of HIV reduces HIV-related mortality and morbidity [3].

ART has transformed HIV-1 from a fatal disease to a chronic disease [4]. But the persistence of HIV in potentially infected cells is a major obstacle to treatment [5]. ART must be taken lifelong, with infected cells having a half-life of 43.9 months, which makes them very resistant to ART [6]. Because of the steady rise of drug-resistant HIV-1 strains and the issue of treatment toxicity, further research into additional ways to control HIV-1 infection is needed [7,8]. Furthermore, discontinuation of therapy can certainly lead to viral rebound that is due to cells harbouring HIV-1 DNA integrated into the host genome [9,10]. Thus, suppressive lifelong ART alone does not conclusively address the HIV pandemic [11]. The long-term goal of HIV treatment is to enable HIV + patients to stop lifelong ART by developing strategies to eradicate cells that are likely to be infected with HIV. Therefore, a better understanding of the mechanisms that regulate HIV-1 infection is essential for intervention in HIV persistent state and for the development of therapeutic strategies.

There is evidence that the initial antiviral immune response may also regulate the establishment and persistence of the viral reservoir [12,13]. CD4 T cells are central to host immunity by providing help to other components of the immune system [14]. CD4 T-cell responses are protective against various pathogenic infections including HIV [15]. With increasing reports of severe immunodeficiencies, CD4 cell counts become a critical part of the care of HIV + individuals [16]. Recent studies have found that non-HIV specific, TCR-activated CD8 + T cells suppress HIV transcription through immune regulatory mechanisms [17]. Tissue resident memory CD8 + T cells predominate and may be critical for maintaining control of HIV replication [18].

Several epigenetic changes, particularly DNA methylation of genes, have been described in HIV transcriptional silencing and have been explored as targets for HIV-1 latency reversal strategies [19]. DNA methylation is a synthetic, reversible, and heritable epigenetic mark, and DNA methylation of CpG dense zones at gene promoters is often associated with direct or indirect transcriptional repression, termed CpG islands [20]. Clinical features, such as timing of infection and duration of antiretroviral therapy, have all been positively associated with accumulation of HIV-1 promoter methylation [21,22].

To further understand the role of gene expression and methylation modifications in HIV + patients, we performed bioinformatics analysis of HIV-associated sequencing data from public databases. Identification of potential targets relevant for diagnosis and treatment of HIV + patients.

## Materials and methods

### Data sources

The HIV data were collected from gene expression omnibus (GEO) databases. GSE29429 included gene expression profiles of whole blood from acute HIV-positive individuals and uninfected controls patients. The samples were sequenced on two platforms by array, GPL6947 and GPL10558. In the GPL6947, there were 147 HIV-positive individuals (87 un-treatment and 60 ART) and 38 uninfected controls. In the GPL10558, there were 30 HIV-positive individuals and 17 uninfected controls. GSE33580 included gene expression profiles of whole blood 43 HIV-resistant and 43 HIV-negative women based on GPL570 by array. The data of GSE29429 and GSE33580 were analysed using lumi R package for normalization method. GSE119234 included gene expression profiles of eight different B-cell subsets which sorted from lymph nodes of 20 HIV- and 31 HIV + individuals based on GPL21697 by high-throughput sequencing. Raw data were background subtracted and normalized as performed by the DEseq2 package of Bioconductor. GSE67748 included DNA methylation profiles of cerebellum from eight HIV + and 12 HIV- human subjects. We used chAMP R package to generate the normalized beta values.

### Differential analysis

The differential analysis between the HIV + patients and controls (or ART and untreatment HIV + patients) was performed by the Limma R software package. The differentially expressed genes (DEGs) were defined as genes with a *p* value <.05. The Limma package was used to obtain the differentially methylated CpG sites with adjusted *p* value <.05.

### Construction of networks

The coexpression network for selected DEGs was performed using Weighted correlation network analysis (WGCNA) by “WGCNA” R package. The soft-thresholding power that we chose was used as the correlation coefficient threshold. Then built a minimum number of genes in modules. The expression pattern of eigengene in each module is condensed into "module eigengene (ME)". Genes in MEs were considered had similar expression patterns.

Through placing module genes into The STRING (Search Tool for Retrieval of Interacting Genes/Proteins), the protein-protein interaction (PPI) network was constructed by screening scores greater than 900. PPI network was displayed through Cytoscape software. Genes were ranked by their degree of connectivity in the network.

### Enrichment analysis

To examine Gene Ontology (GO) and Kyoto Encyclopaedia of Genes and Genomes (KEGG) for module genes, the clusterProfiler R software package was used to perform enrichment analysis. The biological process (BP) was a kind of GO. The R package clusterProfiler was used to obtain the background set for gene set enrichment analysis (GSEA). GSEA runs in Java environment and conducted between HIV + and HIV- subtypes. A *p* value <.05 was considered statistical significance. Single-sample GSEA (GSVA) was carried out using the GSVA package. For each sample, a score for the enrichment of a set of genes using gene expression profile was obtained.

### Single sample gene set enrichment analysis (ssGSEA)

The infiltration level of immune cell was calculated by ssGSEA in GSVA R software package. We analyzed the infiltration of immune cells between HIV + patients and controls (or ART and untreatment HIV + patients). *p* Value <.05 was considered significant.

### LASSO regression analysis

The least absolute shrinkage and selection operator (Lasso) Binomial regression was building using glmnet R package [23]. When performing lasso regression, we retained potential predictors with non-zero coefficients. The optimal lambda value that corresponded most accurate value of cross validation errors was determined to identify potential predictors. The area under the curve (AUC) were performed using pROC R package.

### Sample collection

Peripheral blood samples of 10 persons with primary HIV+, 10 patients under ART and 10 age-matched healthy controls were collected from the Fourth People’s Hospital of Nanning and peripheral blood samples of 10 persons with primary HIV+, 10 patients under ART and 10 age-matched healthy controls were collected from Guiping People's Hospital. All patients gave written informed consent. The human study was approved by the local research ethics committees of Nanning Fourth People's Hospital and Guiping people's Hospital. The study conformed to the Declaration of Helsinki principles.

### Quantitative real-time PCR (qRT-PCR)

The total RNA was extracted from peripheral blood using TRIzol reagent (Invitrogen). Total RNA was reverse transcribed into cDNA using RevertAid RT kit (Thermo Fisher). The cDNA was amplified qRT-PCR using Applied Biosystems™ PowerUp™ SYBR™ Green mix (Thermo Fisher). The primer sequences were shown in Table S1. Relative gene expression was calculated using the 2^−ΔΔCT^ method, with β-actin as the reference gene.

## Results

### Differentially expressed genes in HIV + and HIV-

**Figure 1.**
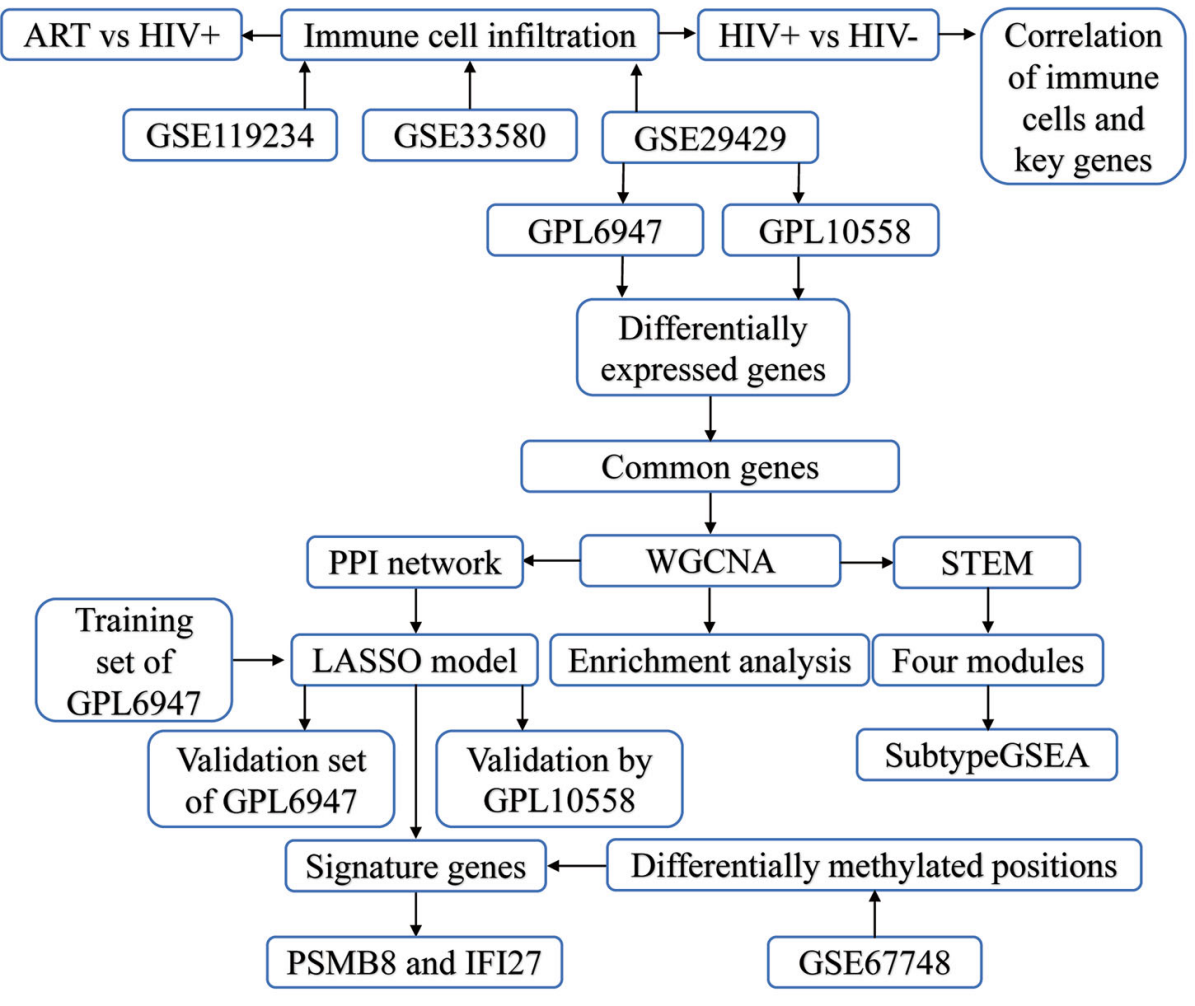
The flowchart of this study.

**Figure 2.**
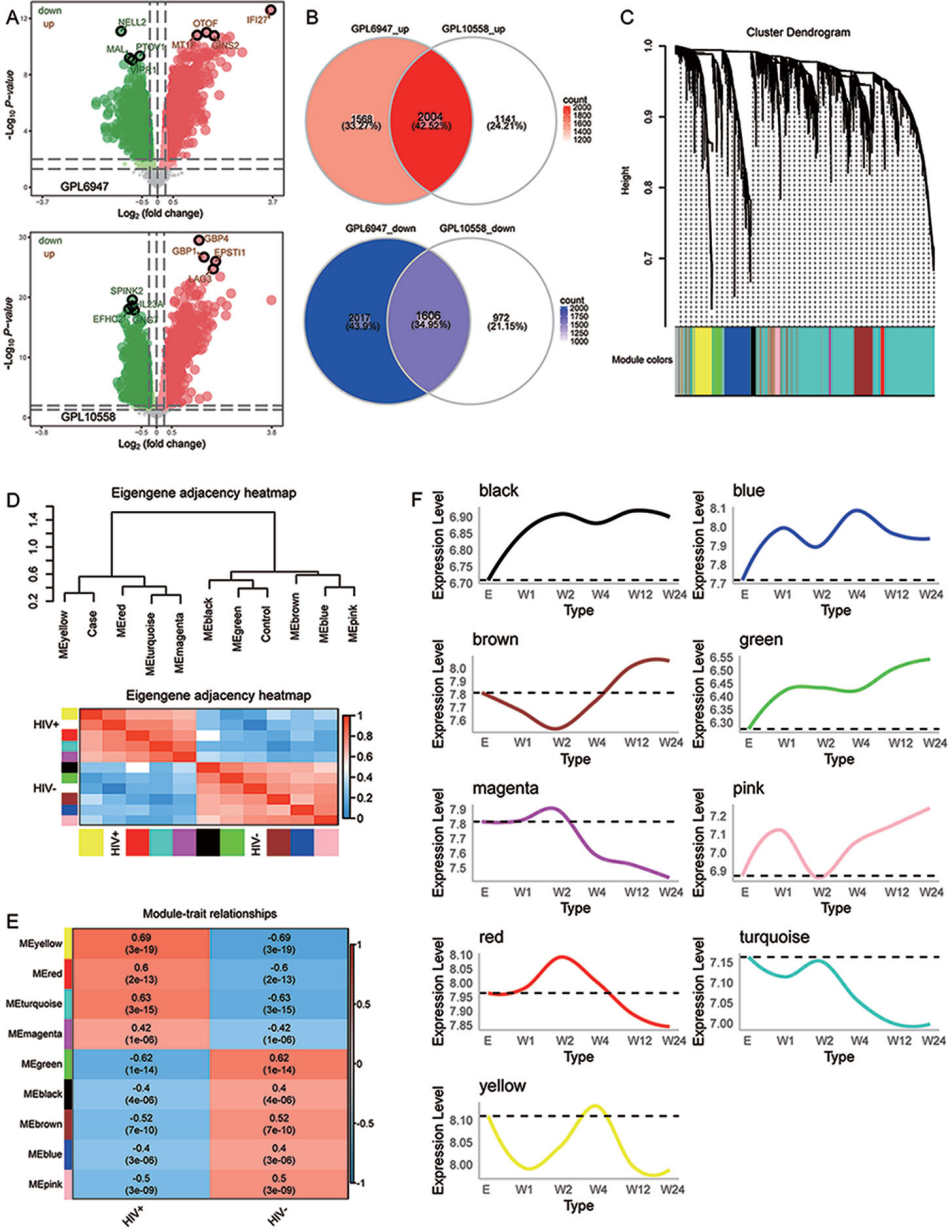
Synergistic expression behaviour of HIV-associated genes. (A) Volcano plot of differentially expressed genes in GPL6947 or GPL10558. (B) Common genes that were up – or down-regulated simultaneously in both sets of DEGs. (C) Clustering tree of coexpression module genes. (D) Eigengene adjacency heatmap of the strength of correlation between modules and clinical trait. (E) Heatmap of correlation between module and clinical phenotype. Red represents positive correlation and blue represents negative correlation. Each row represents a module and each column represents a clinical trait. (F) Module expression changes with time of enrolment after HIV infection. Module expression changes with time of enrolment after HIV infection.

The flowchart of this study is shown in Figure 1. To obtain gene expression signatures associated with HIV + patients, we performed differential analysis of sequencing data in GPL6947 and GPL10558 in GSE2942, respectively. A total of 7195 differentially expressed genes (DEGs) were obtained in GPL6947 and 5723 DEGs were obtained in GPL10558 (Figure 2(A)). Among them, we found 3610 common genes that were up- or down-expressed simultaneously in both sets of DEGs (Figure 2(B)). Further, WGCNA was utilized to explore the coexpression behaviour of common genes. We identified nine coexpression modules (Figure 2(C)). Correlation analysis between modules and clinical traits was performed using Pearson's correlation method, and modules were clustered into two subclasses (Figure 2(D)). Of these, MEyellow (module 7) had the strongest positive correlation with HIV+ (Figure 2(E)). The modules showed different trends of up- or down-regulation with increasing time after enrolment (confirmed acute) of HIV infection (Figure 2(F)).

### Biological functions of module genes enrichment

**Figure 3.**
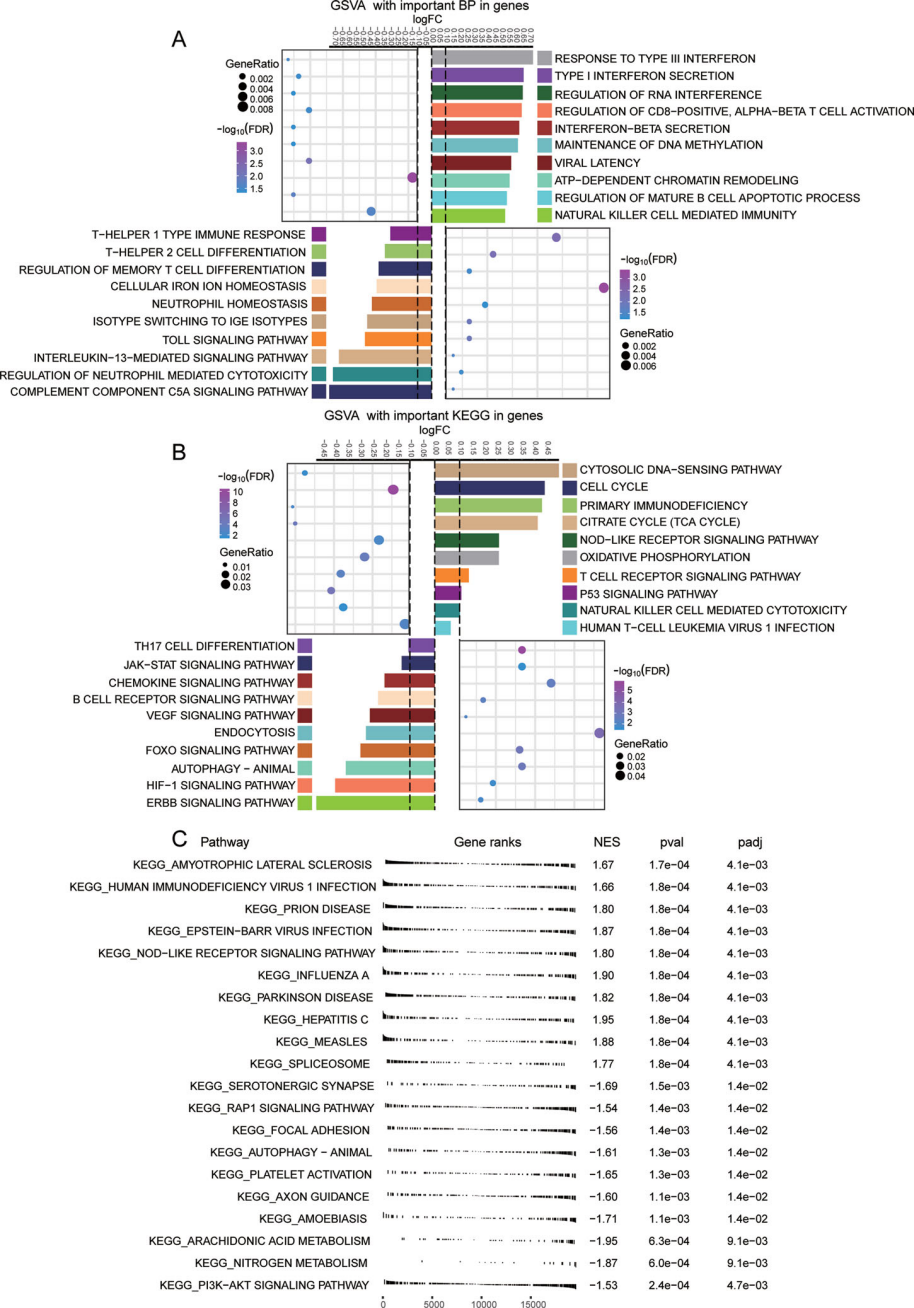
Biological functions and signalling pathways in which module genes participate. (A) Module genes significantly involved in the main up- or down-regulated biological processes. (B) Module genes significantly involved in the main up – or down-regulated KEGG pathways. (C) KEGG in GSEA of module gene involvement.

Enrichment analysis of module genes indicated that they were mainly involved in upregulated biological progression of “response to type III interferon”, “regulation of RNA interference”, and “interferon-β secretion”; downregulated “Toll signalling pathway”, “Th 2 cell differentiation”, and “Th1 type immune response” (Figure 3(A)). As well as the up-regulated “cell cycle”, “NOD-like receptor signalling pathway”, and “primary immunodeficiency” KEGG pathways; the down regulated “autophagy animal”, “Th17 cell differentiation”, and “HIF-1 signalling pathway” KEGG terms (Figure 3(B)). GSEA results also exhibited module genes involved up- or down-regulated KEGG terms (Figure 3(C)). Including up-regulated human immunodeficiency viral 1 infection, NOD-like receptor signalling pathway, and Epstein–Barr viral infection. Down-regulated PI3K − Akt-signalling pathway, autophagy-animal, and Rap1-signalling pathway.

### Genes with persistent expression changes

**Figure 4.**
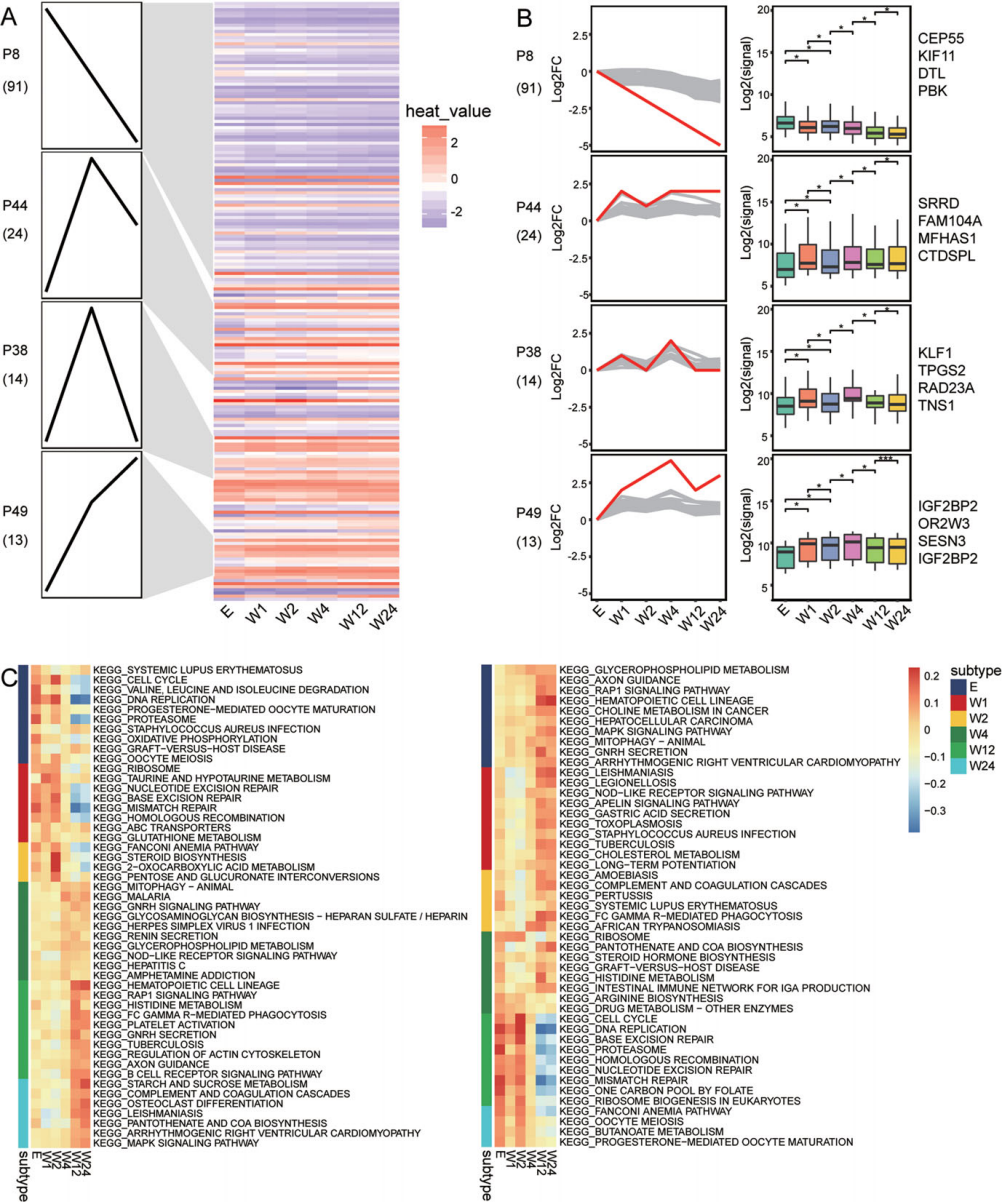
Persistent changes in genes and pathways after HIV enrolment. (A) Heatmap of gene with persistent up- or down-expression from enrolment to 24 weeks of HIV infection. (B) The box plots of STEM genes in four clusters. (C) Signalling pathways that are consistently up- or down-regulated from enrolment to 24 weeks of HIV infection. E: enrolment; W1-24: weeks at 1, 2, 4, 12, and 24 after enrolment.

As the time after enrolment of HIV infection, the expression of genes may become persistently dysregulated. Using STEM software analysis, we obtained 142 genes from coexpression module genes with consistently dysregulated expression (*p* < .05). These genes clustered into distinct modules according to changes in expression trends (Figure 4(A,B)). SubtypeGSEA results showed that MAPK-signalling pathway, complement and coagulation cascades, and starch and sucrose metabolism were consistently up-regulated; homologous recombination, Fanconi anaemia pathway, and butanoate metabolism were consistently down-regulated in the period after HIV infection (Figure 4(C)).

### Identification of key genes

**Figure 5.**
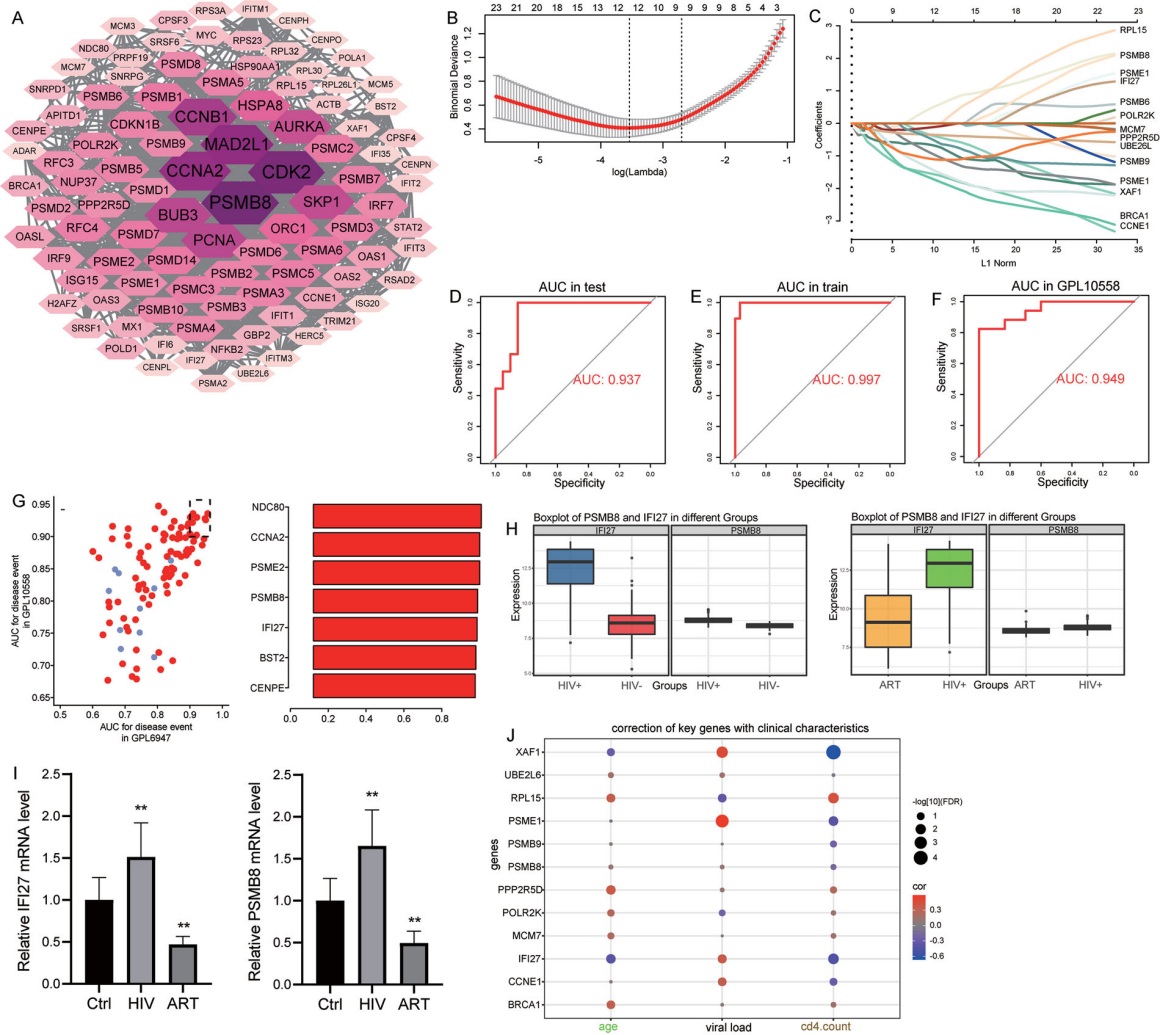
PPI network analysis of module genes identifies key genes. (A). Top 100 most connected genes in the PPI network. The larger the node, the greater the degree. (B). Selection of optimal parameter (*λ*) in the LASSO model. The log(*λ*) value of 12 is used for further analysis. (C) LASSO coefficient profiles of 12 signature genes. (D) AUC values of the signature genes in the training set. (E) AUC values of the signature genes in the validation set. (F) AUC values of the signature genes in GPL10558. (G) AUC values of important genes. (H) Expression levels of PSMB8 and IFI27 in ART or HIV-positive individuals of GPL6947. (I) Relative expression levels of PSMB8 and IFI27 in blood samples of controls, HIV-positive individuals, and ART patients detected by qRT-PCR. **p Value <.01. Ctrl: controls; ART: antiretroviral therapy. (J) Correlations between key genes and clinical features.

To identify the significant genes in the module genes, we performed PPI network analysis. The top 100 genes with the highest degree of connectivity in the PPI network were identified as important genes (Figure 5(A)). We randomly selected one-fourth of the HIV + samples in GPL6947 as training set and the remaining three-fourth as validation set for LASSO region analysis. We then construct a diagnostic model based on 12 signature genes: PSMB8 (proteasome 20S subunit beta 8), POLR2K (RNA polymerase II, I and III subunit K), PSMB9 (proteasome 20S subunit beta 9), PPP2R5D (protein phosphatase 2 regulatory subunit B'delta), PSME1 (proteasome activator subunit 1), CCNE1 (cyclin E1), BRCA1 (BRCA1 DNA repair associated), RPL15 (ribosomal protein L15), XAF1 (XIAP-associated factor 1), IFI27 (interferon alpha inducible protein 27), MCM7 (minichromosome maintenance complex component 7), and UBE2L6 (ubiquitin conjugating enzyme E2 L6) (Figure 5(B,C)). Signature genes had an AUC value of 0.937 in the training set (Figure 5(D)). The AUC value was 0.997 in the validation set (Figure 5(E)). Importantly, the potential diagnostic role of these signature genes was validated by an external dataset, GPL10558 (AUC value was 0.949) (Figure 5(F)). These genes were considered key HIV-associated genes. On the other hand, we obtained genes with AUC values greater than 0.9 in both GPL6947 and GPL10558 (Figure 5(G)). Among them, PSMB8 and IFI27 were also signature genes. Surprisingly, IFI27 was present in STEM results and became progressively dysregulated genes as HIV progresses. In addition, PSMB8 and IFI27 showed decreased expression levels after ART (Figure 5(H)). This was also confirmed by the results of qRT-PCR experiments in clinical blood samples (Figure 5(I)). Correlation analysis showed opposite levels of correlation between viral load and CD4 count with key genes (Figure 5(J)). Suggesting that key genes may be relevant for the treatment of HIV.

### Identification of methylation marks

**Figure 6.**
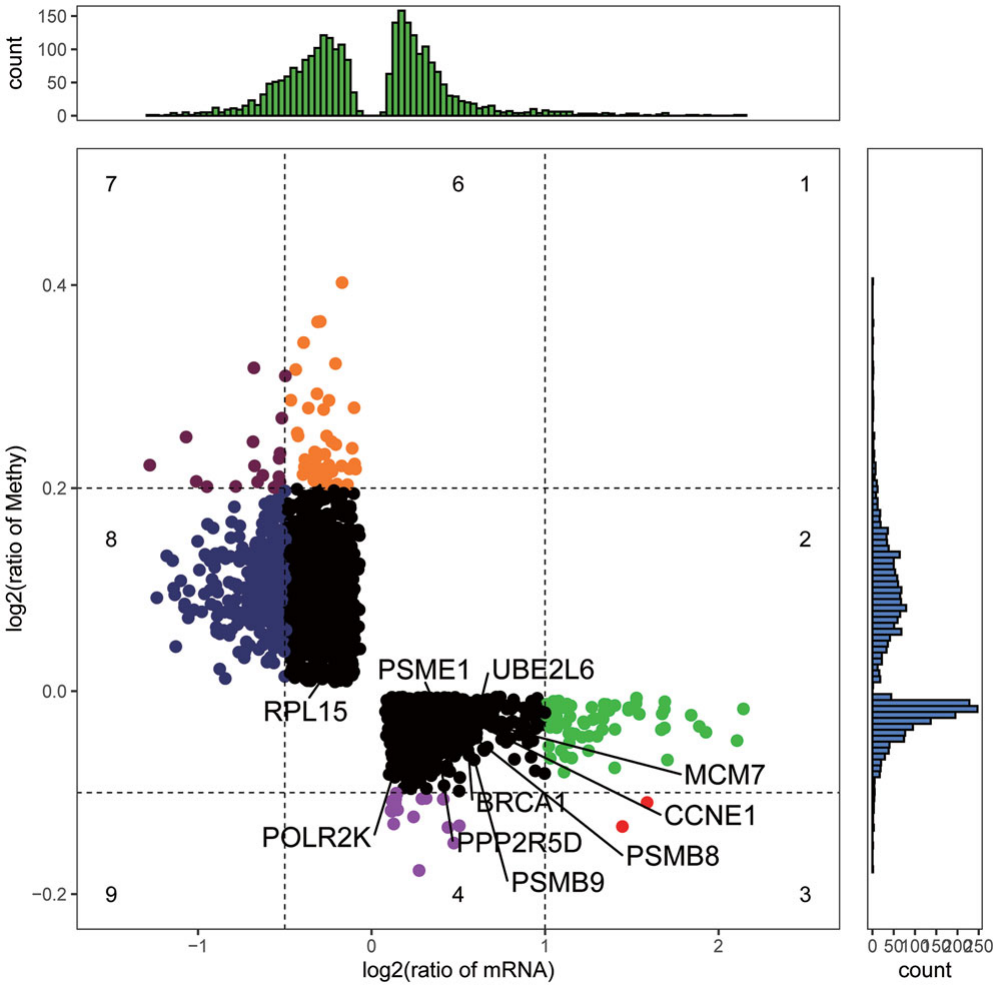
Methylation and expression levels of methylation marks.

To identify methylation modifications during HIV development, we performed differential analysis of GSE67748. Then, 216136 differentially methylated positions (DMPs) were found between HIV + and HIV- patients (Figure S1). Which included 85.65% hypomethylated DMPs and 14.35% hyermethylated DMPs. Genes were considered as methylation marks when their deltabeta values and logFC values were in opposite directions. After comparison with common genes, we found 2647 methylation marks (Figure 6). PSMB8, POLR2K, PSMB9, PPP2R5D, PSME1, CCNE1, BRCA1, RPL15, MCM7 and UBE2L6 of key genes were all subject to methylation modification.

### Immune cell infiltration in HIV infection

**Figure 7.**
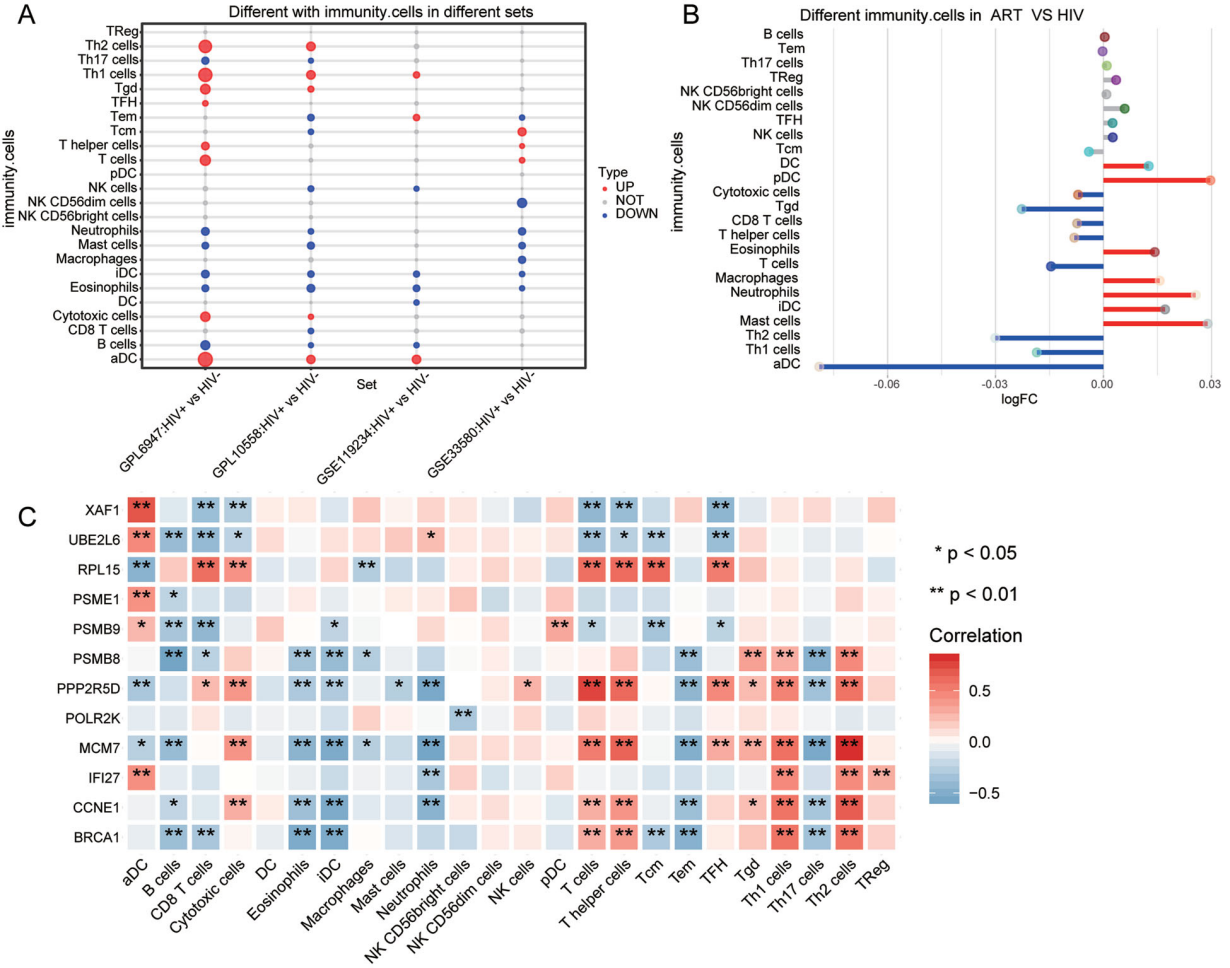
Immune cell infiltration levels in HIV + patients. (A) Differences in immune cell infiltration between HIV + and HIV-patients in the four datasets. (B) Differences in immune cell infiltration between ART and HIV + patients. (C) Correlation between immune infiltrating cells and key genes.

Immune cell expression was calculated for each sample by ssGSEA and compared for differences in immune cell infiltration between HIV + and HIV- patients in GPL6947, GPL10558, GSE119234, and GSE33580. Th1 cells and activated dendritic cells (aDCs) showed significantly up-regulated expression in all three datasets, Eosinophils, iDC, Mast cells, Neutrophils and B cells were significantly down-regulated (Figure 7(A)). The infiltration of immune cells was altered when HIV + patients received ART (Figure 7(B)). This showed that ART was associated with infiltration of immune cells. We then calculated the correlation between key genes and immune cells in HIV + patients (Figure 7(C)). There was a significant positive correlation between Th1 cells, Th2 cells, and aDC with IFI27.

## Discussion

The aim of this study was to investigate gene expression patterns in HIV + patients, identify candidate gene biomarkers, and complex mechanisms during the course of the disease. Attempts were made to screen for potential therapeutic targets to halt exacerbations in HIV-positive individuals. We constructed a coexpression network by taking advantage of gene expression differences between HIV + and HIV- patients in the GEO database. Identified gene sets associated with the course of HIV + patients, as well as persistently dysregulated signalling pathways. Further utilization of the PPI network and LASSO model identified key genes, potentially involved in the control of viral replication. In addition, the expression of some key genes is modified by methylation, which may be an important means of regulating the progression of HIV infection.

We performed differential analysis of the sequencing data from the two data platforms and obtained DEGs that were up- or down-regulated simultaneously, so that the obtained gene sets might be more relevant to HIV. WGCNA builds network models that rely on statistical methods, improves simple correlation networks, and quantifies the extent to which these genes have identical neighbours [24,25]. WGCNA provides an effective method to identify gene sets with similar expression for correlation with phenotype [26]. In the results of our analysis, the expression trends of different modules differed with longer time after enrolment of HIV infection. This implies that module genes may be associated with the course of HIV. Further using STEM software, we obtained consistently expressed dysregulated genes significantly associated with HIV progression. These genes may influence HIV exacerbation.

Based on the enrichment analysis of module genes, we obtained the biological functions that were gradually up- or down-regulated as the time after HIV infection was extended. Interferons (IFNs), which control HIV-1 replication, produce a marked and transient reduction in plasma viral load [27]. Post-HIV-1 exposure elicits a defensive role of the innate immune system, including interferon signalling, one of the main effectors [28]. Studies have confirmed that Th1/17 polarization enriched CD4 T cells have higher susceptibility to HIV-1 infection in *in vitro* and *in vivo* experiments [29]. Studies have reported the ability of nucleotide binding oligomerization domain (NOD)-like receptors (NLRs) in platelets to sustain viral infection and replication, including HIV-1 [30]. On the other hand, NLRP3 is an NLR family member whose activation within microglia is an important mechanism by which cells exert anti-HIV [31]. The regulation of HIV-1 infection by NLRs needs more in-depth study. The viral protein Vpr of HIV promotes HIF-1 α expression by activating cellular oxidative stress, which in turn promotes the transcription of viral genes [32]. In the results of our analysis, up- or down-regulation of biological functions, both related to the host immune inflammatory response after HIV infection.

The results of our analysis reconfirmed the differences in immune cell infiltration levels between HIV + and HIV- patients [33,34]. Dendritic cells (DCs) are the first HIV virus-exposed immune cells linking innate and adaptive immune responses, holding promise for functional treatment of HIV-1 infection [35]. Physiological proliferation of HIV-1-infected Th1 cells plays a crucial role in supporting the persistence of HIV-1 [36]. Extensive communication of mast cells with other types of cells during viral infection may provide more opportunities to halt viral spread [37,38]. Intriguingly, mast cells are thought to contribute to persistent HIV-1 infection [39,40].

The key genes that we identified had elevated expression in patients with HIV infection and decreased expression in patients on ART. Suggesting that these genes may be relevant for the treatment of HIV. Among them, IFI27 was found to be strongly correlated with Th1 cells, Th2 cells and aDC, positively correlated with viral load, and also negatively correlated with CD4 count. These results suggested that IFI27 expression may be involved in exacerbation of HIV + patients and was a potential therapeutic target. Up-regulation of interferon (IFN) – alpha inducible protein 27 (IFI27) may be associated with inflammatory events [41]. It has been confirmed that IFI27 was expressed in higher amounts in HIV + patients than in HIV- patients and positively correlated with the viral load of HIV-1 [42,43]. Suggested that IFI27 may contribute to the mechanism of immunodeficiency, HIV replication, in HIV-1 disease.

In addition, we found that 10 key genes were subjected to low-level methylation modification. DNA methylation of HIV-1 promoter/enhancer sequences plays an important role in its maintenance [21]. Most significant CPGs are hypomethylated in HIV + individuals compared to HIV- individuals [44]. Study has shown that HIV viral proteins are highly involved in the complex interplay of chromatin rearrangements and that methyltransferases are involved in this process [45]. DNA methylation, as a regulatory mechanism of host genes involved in immune HIV-1 control, may have effectiveness in interfering with therapeutic strategies [46,47].

This study has several limitations. First, our results must be confirmed in a larger sample before they can be applied to the clinic. Second, the sample information of data obtained from public databases was still limited, which may have limited the accuracy of results interpretation to some extent. In addition, we only screened key genes for their association with the timing of HIV infection, with immune cells and viral load, but there is a paucity of relevant studies on how key genes regulate the disease course of HIV. Especially, the data of the effect of IFI27 on HIV-1 replication experiment in cells will be performed in the future. In conclusion, there is still a long way to go to fully unravel the biological processes and potential targets of HIV using bioinformatics methods.

## Conclusion

In summary, in this study, WGCNA was used to screen key genes associated with HIV infection using a combination of enrichment analysis and LASSO modelling. IFI27 was identified to be associated with the timing of HIV infection and ART, revealing its importance in the alteration of the immune environment during HIV infection, suggesting that IFI27 may be a potential therapeutic target for HIV. The results of our analysis provided new markers for understanding the molecular mechanisms underlying the progression of HIV infection and for facilitating the exploration of therapeutic targets.

## Data Availability

The data that support the findings of this study are available from the corresponding author, upon reasonable request.
